# Optimal time and threshold of absolute lymphocyte count recovery as a prognostic factor after single‐unit cord blood transplantation in adults

**DOI:** 10.1002/jha2.372

**Published:** 2021-12-29

**Authors:** Takaaki Konuma, Maki Monna‐Oiwa, Kosuke Takano, Masamichi Isobe, Seiko Kato, Satoshi Takahashi, Yasuhito Nannya

**Affiliations:** ^1^ Department of Hematology/Oncology The Institute of Medical Science The University of Tokyo Tokyo Japan; ^2^ Division of Clinical Precision Research Platform The Institute of Medical Science The University of Tokyo Tokyo Japan

**Keywords:** absolute lymphocyte count, allogeneic hematopoietic cell transplantation, cord blood transplantation, immune reconstitution, non‐relapse mortality, survival

## Abstract

We retrospectively evaluated the optimal time and threshold of absolute lymphocyte count (ALC) recovery as a prognostic factor in 174 adult patients who received single‐unit cord blood transplantation (CBT) at our institute. We analyzed the impact of ALC ≥300, ≥600, and ≥900/μl by 30 and 60 days on transplant outcomes. Multivariate analysis showed that only ALC ≥300/μl at 60 days was significantly associated with overall mortality (hazard ratio, 0.24; *p* = 0.001) following CBT. The optimal time point to use ALC recovery as a prognostic tool following CBT could be later than those following adult donor transplantation.

## 1 INTRODUCTION

Delayed immune reconstitution is one of the major limitations of cord blood transplantation (CBT). Previous studies clearly demonstrated that absolute lymphocyte count (ALC) recovery, which may be a useful surrogate marker of immune reconstitution, predicted survival following CBT [1, 2, 3, 4] as well as allogeneic hematopoietic cell transplantation (HCT) from adult donors [5, 6]. However, various thresholds and time points of ALC recovery following CBT have been reported to be prognostic factors for CBT [1, 2, 3, 4]. Therefore, we evaluated the optimal time and threshold of ALC recovery as a prognostic factor following CBT.

## 2 METHODS

We included 174 consecutive adult patients who underwent single‐unit CBT as a first allogeneic HCT at our institute between March 2007 and December 2020. The selection of cord blood unit, conditioning regimen, graft‐versus‐host disease (GVHD) prophylaxis, and supportive care were determined by the treating physicians, as previously described [7, 8, 9, 10, 11, 12]. No patients received antithymocyte globulin, alemtuzumab, or rituximab as a conditioning regimen, or GVHD prophylaxis. For evaluation of ALC recovery, complete blood counts using an automated hematology analyzer (XE‐2100; Sysmex, Kobe, Japan) and manual differential leukocyte counts were evaluated at least three times per week from the day of neutrophil recovery to 60 days following CBT. We analyzed the impact of ALC ≥300, ≥600, and ≥900/μl by 30 and 60 days on transplant outcomes. The institutional review board of our institute approved this retrospective study (2021‐60‐1110).

Statistical analyses were calculated using EZR (Saitama Medical Center, Jichi Medical University, Saitama, Japan) [13], a graphical user interface for the R 4.1.1 software program (R Foundation for Statistical Computing, Vienna, Austria). Overall survival (OS) was defined as the time from CBT to death or last contact. Relapse was defined as the presence of hematological disease as an indication for CBT. Non‐relapse mortality (NRM) was defined as death without relapse. The probability of OS was calculated using the Kaplan–Meier method, and the cumulative incidence function was used to estimate ALC recovery, relapse, and NRM to accommodate competing risks. Univariate analyses were performed using a log‐rank test for OS and Gray's test for relapse and NRM with a landmark analysis at 30 or 60 days after CBT, because ALC recovery was evaluated at 30 or 60 days after CBT. The competing risk for relapse was NRM, whereas the competing risk for NRM was relapse. For ALC recovery, death before 30 or 60 days following CBT was a competing event.

Multivariate analyses were performed using a Fine and Gray model for ALC recovery and the Cox proportional hazards model for overall mortality, relapse, and NRM. In the Cox proportional hazards models, ALC recovery and corticosteroid therapy were treated as time‐varying covariates, and patients who experienced relapse or NRM were censored for evaluation of relapse and NRM. The following covariates, other than ALC recovery and corticosteroid therapy, were considered in the multivariate analysis: age (<45 vs. ≥45 years), recipient sex (male vs. female), refined disease risk index (low/intermediate vs. high/very high) [14], cryopreserved cord blood CD34^+^ cell dose (<1 × 10^5^ vs. ≥1 × 10^5^/kg), HLA disparities defined as a high‐resolution for HLA‐A, ‐B, and ‐DRB1

(<3 vs. ≥3), and GVHD prophylaxis (cyclosporine and methotrexate [CSP + MTX] vs. CSP + MMF [mycophenolate mofetil]). Age and cryopreserved CD34^+^ cell dose were divided according to approximately median values, and corticosteroid therapy was defined as systemic administration equivalent to 1 mg/kg/day or more prednisolone within the first 30 or 60 days following CBT. To adjust for multiple testing for each outcome in multivariate analysis, *p* < 0.00833 (0.05/6) was considered statistically significant with the Bonfferoni correction. *p* values between 0.00833 and 0.05 were considered to have a marginal significance.

## 3 RESULTS

Patient characteristics are shown in Table 1. The median age was 45.5 years. The most common disease type was acute myeloid leukemia in 87 patients (50%). The most common GVHD prophylaxis was CSP + MTX (78%). The median cryopreserved cord blood total nucleated cell (TNC) dose and CD34^+^ cell dose were 2.58 × 10^7^/kg and 1.02 × 10^5^/kg, respectively. The median follow‐up for survivors was 5.2 years (range, 0.2–13.0 years). CSP + MMF for GVHD prophylaxis was significantly associated with older age, higher disease risk index, and other than total body irradiation ≥10 Gy‐based regimens. GVHD prophylaxis significantly affected OS, NRM, and grades III–IV acute GVHD in univariate analysis (Figure S1).

**TABLE 1:** Patients and transplantation characteristics

Characteristics	Value
Number of patients	174
Median age at CBT, (range) years	45.5 (16–69)
Sex	
Male	109 (63%)
Female	65 (37%)
Recipients CMV serostatus	
Positive	146 (84%)
Negative	28 (16%)
Diagnosis	
AML	87 (50%)
ALL	36 (21%)
MDS	26 (15%)
MPN/ CMML	7 (4%)
NHL/ATL	7 (4%)
CML	6 (3%)
CAEBV/ SAA	5 (3%)
Refined disease risk index	
Low/Intermediate	85 (49%)
High/Very high	83 (48%)
Not available	6 (3%)
Conditioning regimen	
TBI ≥10 Gy‐based regimens	137 (79%)
Others	37 (21%)
GVHD prophylaxis	
CSP with MTX	136 (78%)
CSP with MMF	38 (22%)
Cryopreserved TNC dose, (range) x10⁷/kg	2.58 (1.52–5.69)
Cryopreserved CD34⁺ cell dose, (range) x10⁵/kg	1.02 (0.36–2.84)
HLA disparities	
<3	82 (47%)
≥3	92 (53%)

Abbreviations: ALL, acute lymphoblastic leukemia; AML, acute myeloid leukemia; ATL, adult T‐cell leukemia; CAEBV, chronic active Epstein‐Barr virus infection; CBT, cord blood transplantation; CML, chronic myelogenous leukemia; CMML, chronic myelomonocytic leukemia; CMV, cytomegalovirus; CSP, cyclosporine; GVHD, graft‐versus‐host disease; HLA, human keukocyte antigen; MDS, myelodysplastic syndrome; MMF, mycophenolate mofetil; MPN myeloproliferative neoplasm; MTX, methotrexate; NHL, non‐Hodgkin's lymphoma; SAA, severe aplastic anemia; TBI, total body irradiation; TNC, total nucleated cell.

HLA disparities were defined as a high‐resolution for HLA‐A, ‐B, and ‐DRB1.

**FIGURE 1:**
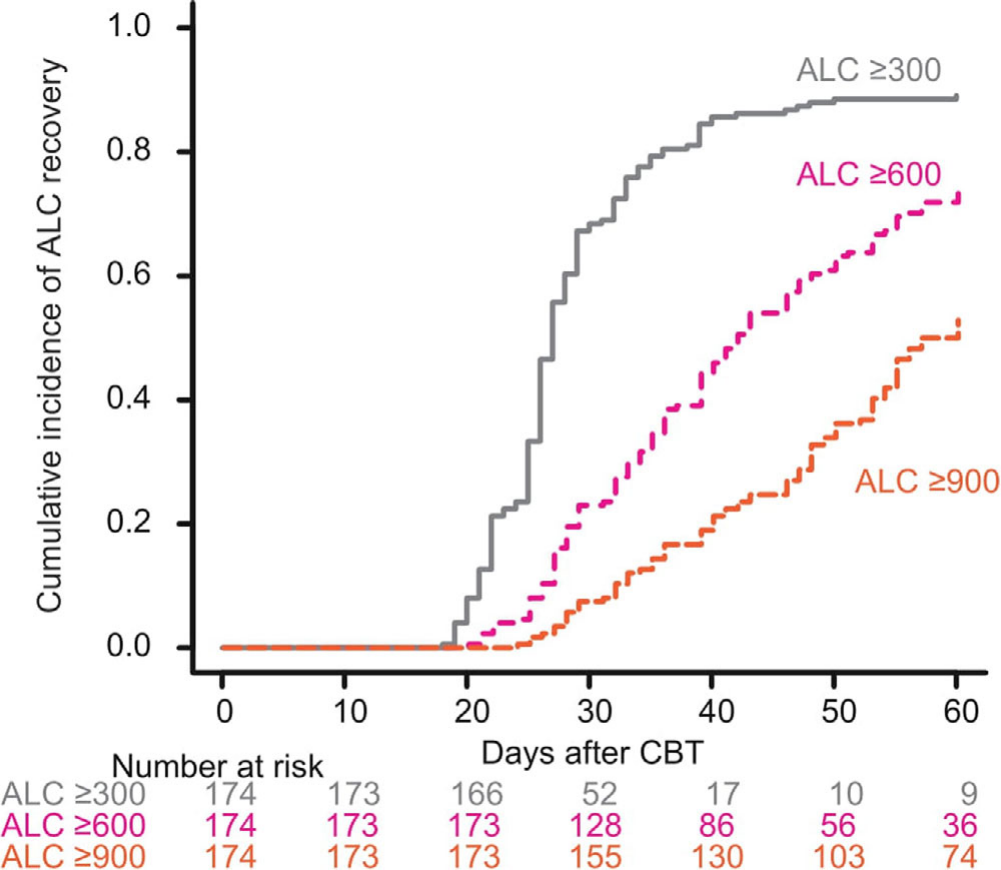
The cumulative incidence of absolute lymphocyte count (ALC) recovery after cord blood transplantation (CBT)

The cumulative incidences of ALC recovery to ≥300, ≥600, or ≥900/μl at 30 days were 68% (95% confidence interval [95% CI]: 61%–75%), 23% (95% CI: 17%–30%), and 8% (95% CI: 4%–12%), respectively. The cumulative incidences of ALC recovery to ≥300, ≥600, or ≥900/μl at 60 days were 89% (95% CI: 83%–93%), 74% (95% CI: 66%–80%), and 53% (95% CI: 45%–60%), respectively (Figure 1).

In the multivariate analysis, higher CD34^+^ cell dose was significantly associated with better ALC recovery ≥300/μl at 30 days (HR: 2.52; 95% CI: 1.77–3.59; *p* < 0.001) and ALC recovery ≥300/μl at 60 days (hazard ratio [HR]: 1.87; 95% CI: 1.35–2.60; *p* < 0.001). Older age was significantly associated with worse ALC recovery ≥600/μl at 60 days (HR: 0.57; 95% CI: 0.38–0.84; *p *= 0.005) (Table 2). We also analyzed the effect of TNC dose using different threshold (≥2.0, ≥2.5, or ≥3.0 × 10^7^/kg). But, TNC dose did not affect the ALC recovery in the multivariate analysis (Table S1). There were no significance differences of incidences of acute GVHD, cytomegalovirus (CMV) antigenemia, virus infections, and bacteremia up to 60 days after CBT between patients with or without ALC recovery ≥300/μl by 60 days, but higher incidences of grades III–IV acute GVHD and CMV antigenemia up to 60 days after CBT were observed in patients without ALC recovery ≥600/μl or 900/μl by 60 days (Table S2).

**TABLE 2:** Multivariable analysis for ALC recovery

	ALC ≥300 /μl		ALC ≥600 /μl		ALC ≥900 /μl	
	Adjusted HR (95% CI)	*p* value	Adjusted HR (95% CI)	*p* value	Adjusted HR (95% CI)	*p* value
By 30 days						
Age ≥45 years vs. <45 years	0.82 (0.56–1.20)	0.320	0.65 (0.34–1.25)	0.200	0.50 (0.15–1.61)	0.250
Female recipient vs. male recipient	1.24 (0.87–1.78)	0.230	1.30 (0.68–2.49)	0.420	0.94 (0.30–2.92)	0.920
Higher rDRI vs. lower rDRI	0.75 (0.52–1.09)	0.140	0.85 (0.44–1.64)	0.640	0.52 (0.15–1.84)	0.310
CD34^+^dose ≥1 × 10^5^/kg vs. <1 × 10^5^/kg	2.52 (1.77–3.59)	**<0.001**	1.96 (1.03–3.72)	0.040	3.46 (0.97–12.27)	0.054
HLA disparities ≥3 vs. <3	1.12 (0.78–1.60)	0.530	0.84 (0.44–1.60)	0.610	0.79 (0.27–2.24)	0.660
CSP + MMF vs. CSP + MTX	0.56 (0.32–0.97)	0.041	0.20 (0.04–0.93)	0.041	0.50 (0.04–5.43)	0.570
By 60 days						
Age ≥45 years vs. <45 years	0.81 (0.57–1.16)	0.260	0.57 (0.38–0.84)	**0.005**	0.45 (0.28–0.73)	0.013
Female recipient vs. male recipient	1.30 (0.95–1.77)	0.095	1.42 (1.00–2.01)	0.047	1.16 (0.75–1.80)	0.490
Higher rDRI vs. lower rDRI	0.79 (0.57–1.08)	0.140	0.72 (0.51–1.03)	0.077	0.67 (0.44–1.04)	0.077
CD34^+^dose ≥1 × 10^5^/kg vs. <1 × 10^5^/kg	1.87 (1.35–2.60)	**<0.001**	1.41 (0.99–2.00)	0.051	1.68 (1.10–2.58)	0.016
HLA disparities ≥3 vs. <3	0.97 (0.71–1.34)	0.900	1.00 (0.71–1.42)	0.980	0.96 (0.64–1.45)	0.870
CSP + MMF vs. CSP + MTX	0.62 (0.40–0.96)	0.035	0.73 (0.42–1.27)	0.270	1.16 (0.58–2.32)	0.660

*Note*: The *p* values in bold are statistically significant (<0.0083).

Abbreviations: ALC, absolute lymphocyte count; CI, confidence interval; CSP, cyclosporine; HR, hazard ratio; MMF, mycophenolate mofetil; MTX, methotrexate; rDRI, refined disease risk index.

**FIGURE 2:**
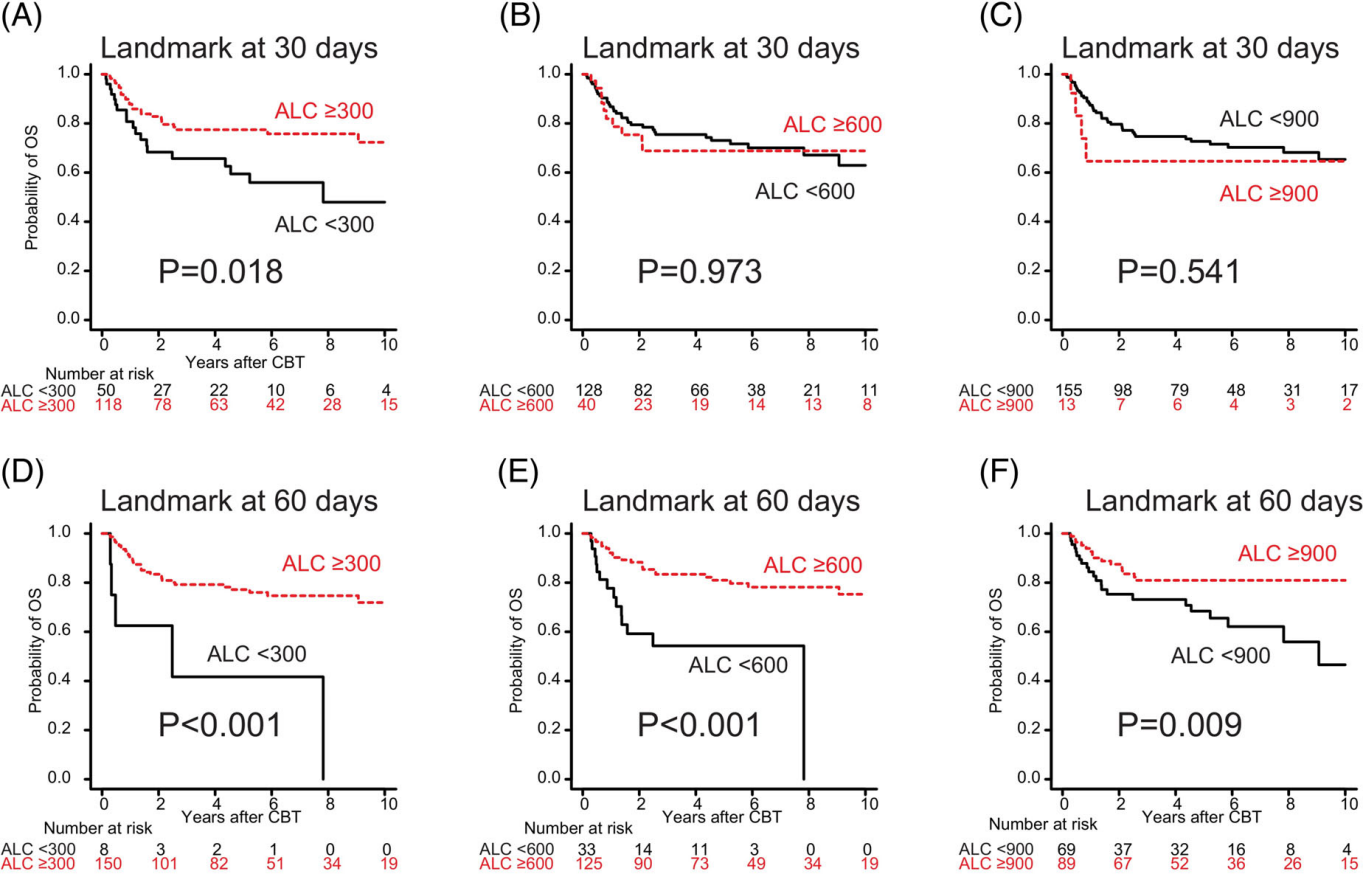
The probability of overall survival (OS) following cord blood transplantation (CBT) according to absolute lymphocyte count (ALC) recovery ≥300, ≥600, or ≥900/μl after 30 and 60 days. Kaplan–Meier survival curves were plotted with a conditional landmark analysis at 30 days (A–C) and 60 days (D–F) following CBT

In the univariate analysis with a conditional landmark analysis at 30 days, ALC recovery ≥300/μl (*p* = 0.018) was significantly associated with better OS. In the univariate analysis with a conditional landmark analysis at 60 days, ALC recovery ≥300/μl (*p* < 0.001), ALC recovery ≥600/μl (*p* < 0.001), and ALC recovery ≥900/μl (*p* = 0.009) were significantly associated with better OS (Figure 2). In the multivariate analysis, only ALC recovery ≥300/μl by 60 days was significantly associated with lower overall mortality (HR: 0.24; 95% CI: 0.10–0.56; *p* = 0.001) (Table 3).

**TABLE 3:** Multivariable analysis for overall mortality

	ALC ≥300/μl		ALC ≥600/μl		ALC ≥900/μl	
	Adjusted HR (95% CI)	*p* value	Adjusted HR (95% CI)	*p* value	Adjusted HR (95% CI)	*p* value
By 30 days						
ALC recovery	0.56 (0.29–1.09)	0.092	1.04 (0.47–2.29)	0.904	0.79 (0.25–2.52)	0.700
Age ≥45 years vs. <45 years	1.87 (0.91–3.81)	0.085	1.93 (0.94–3.97)	0.071	1.92 (0.94–3.91)	0.069
Female recipient vs. male recipient	0.37 (0.18–0.78)	0.009	0.36 (0.17–0.77)	**0.0082**	0.35 (0.16–0.76)	**0.007**
Higher rDRI vs. lower rDRI	2.04 (1.11–3.72)	0.020	2.11 (1.15–3.85)	0.098	2.11 (1.16–3.84)	0.014
CD34^+^dose ≥1 × 10^5^/kg vs. <1 × 10^5^/kg	0.96 (0.50–1.84)	0.917	0.82 (0.44–1.54)	0.554	0.86 (0.45–1.63)	0.652
HLA disparities ≥3 vs. <3	0.70 (0.37–1.31)	0.271	0.69 (0.37–1.31)	0.266	0.69 (0.37–1.30)	0.256
CSP + MMF vs. CSP + MTX	1.65 (0.79–3.44)	0.181	1.85 (0.89–3.85)	0.098	1.83 (0.88–3.78)	0.102
Corticosteroid therapy	6.39 (2.90–14.09)	**<0.001**	5.56 (2.50–12.38)	**<0.001**	5.93 (2.68–13.11)	**<0.001**
By 60 days						
ALC recovery	0.24 (0.10–0.56)	**0.001**	0.51 (0.26–1.00)	0.051	0.62 (0.32–1.19)	0.156
Age ≥45 years vs. <45 years	2.04 (0.99–4.19)	0.052	1.86 (0.89–3.87)	0.096	1.80 (0.85–3.83)	0.122
Female recipient vs. male recipient	0.35 (0.16–0.77)	0.009	0.39 (0.18–0.83)	0.014	0.38 (0.18–0.82)	0.013
Higher rDRI vs. lower rDRI	2.41 (1.29–4.48)	**0.005**	2.11 (1.13–3.94)	0.018	2.16 (1.16–4.02)	0.014
CD34^+^dose ≥1 × 10^5^/kg vs. <1 × 10^5^/kg	0.96 (0.50–1.83)	0.914	0.97 (0.51–1.85)	0.946	0.97 (0.51–1.84)	0.927
HLA disparities ≥3 vs. <3	0.67 (0.36–1.27)	0.228	0.83 (0.45–1.53)	0.562	0.84 (0.45–1.55)	0.579
CSP + MMF vs. CSP + MTX	1.47 (0.69–3.12)	0.314	1.50 (0.71–3.19)	0.282	1.65 (0.77–3.53)	0.189
Corticosteroid therapy	3.42 (1.79–6.53)	**<0.001**	2.88 (1.53–5.43)	**0.001**	3.13 (1.68–5.86)	**<0.001**

*Note*: The *p* values in bold are statistically significant (<0.0083).

Abbreviations: ALC, absolute lymphocyte count; CI, confidence interval; CSP, cyclosporine; HR, hazard ratio; MMF, mycophenolate mofetil; MTX, methotrexate; rDRI, refined disease risk index.

**FIGURE 3:**
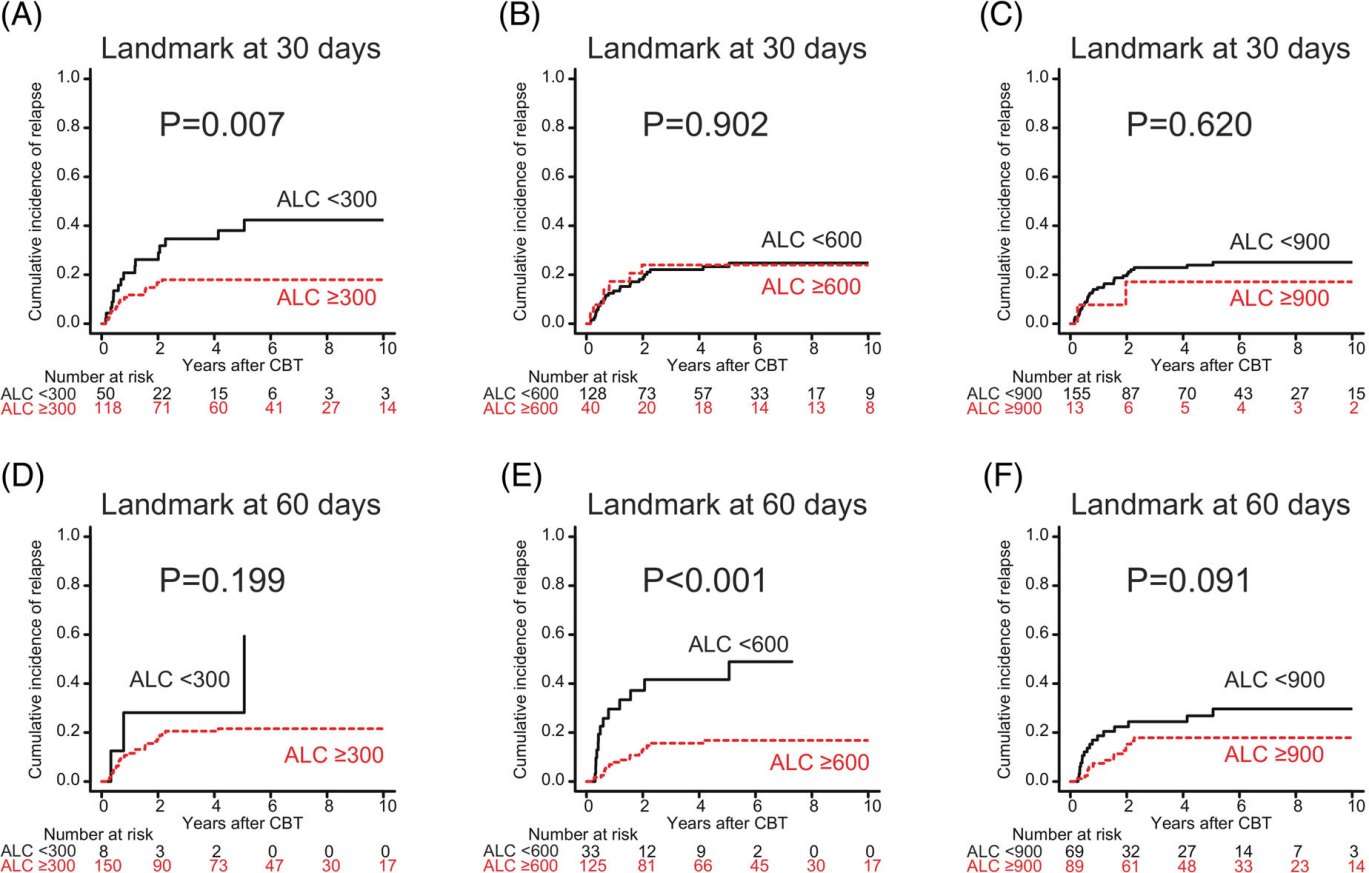
The cumulative incidence of relapse following cord blood transplantation (CBT) according to absolute lymphocyte count (ALC) recovery of ≥300, ≥600, or ≥900/μl by 30 and 60 days. Cumulative incidence curves were plotted with a conditional landmark analysis at 30 days (A–C) and 60 days (D–F) following CBT

In the univariate analysis with a conditional landmark analysis at 30 days, ALC recovery ≥300/μl (*p* = 0.007) was significantly associated with a lower risk of relapse. In the univariate analysis with a conditional landmark analysis at 60 days, ALC recovery ≥600/μl (*p* < 0.001) was significantly associated with a lower risk of relapse (Figure 3). In the multivariate analysis, only ALC recovery ≥600/μl by 60 days (HR: 0.21; 95% CI: 0.09–0.47; *p* < 0.001) was significantly associated with a lower risk of relapse (Table 4).

**TABLE 4:** Multivariable analysis for relapse

	ALC ≥300/μl		ALC ≥600/μl		ALC ≥900/μl	
	Adjusted HR (95% CI)	*p* value	Adjusted HR (95% CI)	*p* value	Adjusted HR (95% CI)	*p* value
By 30 days						
ALC recovery	0.44 (0.21–0.92)	0.031	1.32 (0.56–3.09)	0.511	0.90 (0.20–3.94)	0.898
Age ≥45 years vs. <45 years	1.88 (0.88–4.00)	0.101	2.07 (0.95–4.50)	0.066	1.94 (0.91–4.15)	0.084
Female recipient vs. male recipient	0.87 (0.43–1.79)	0.724	0.79 (0.38–1.64)	0.537	0.82 (0.40–1.69)	0.601
Higher rDRI vs. lower rDRI	4.11 (1.89–8.93)	**<0.001**	4.36 (2.00–9.50)	**<0.001**	4.23 (1.96–9.11)	**<0.001**
CD34^+^dose ≥1 × 10^5^/kg vs. <1 × 10^5^/kg	1.32 (0.63–2.77)	0.458	1.01 (0.50–2.02)	0.971	1.01 (0.50–2.02)	0.970
HLA disparities ≥3 vs. <3	0.74 (0.37–1.48)	0.408	0.71 (0.35–1.41)	0.328	0.71 (0.36–1.42)	0.338
CSP + MMF vs. CSP + MTX	0.33 (0.11–0.96)	0.042	0.38 (0.13–1.12)	0.081	0.37 (0.12–1.08)	0.069
Corticosteroid therapy	0.36 (0.04–2.83)	0.338	0.28 (0.03–2.21)	0.228	0.31 (0.04–2.48)	0.275
By 60 days						
ALC recovery	0.49 (0.13–1.81)	0.290	0.21 (0.09–0.47)	**<0.001**	0.56 (0.27–1.15)	0.118
Age ≥45 years vs. <45 years	1.91 (0.90–4.05)	0.088	1.54 (0.71–3.36)	0.271	1.73 (0.81–3.72)	0.154
Female recipient vs. male recipient	0.79 (0.38–1.65)	0.541	1.02 (0.48–2.18)	0.945	0.79 (0.38–1.63)	0.527
Higher rDRI vs. lower rDRI	4.40 (2.04–9.48)	**<0.001**	4.87 (2.17–10.93)	**<0.001**	4.39 (2.02–9.51)	**<0.001**
CD34^+^dose ≥1 × 10^5^/kg vs. <1 × 10^5^/kg	1.13 (0.56–2.30)	0.717	1.29 (0.63–2.63)	0.473	1.22 (0.59–2.52)	0.572
HLA disparities ≥3 vs. <3	0.67 (0.33–1.35)	0.268	0.73 (0.36–1.46)	0.385	0.70 (0.35–1.40)	0.316
CSP + MMF vs. CSP + MTX	0.37 (0.12–1.09)	0.073	0.25 (0.08–0.77)	0.015	0.35 (0.12–1.04)	0.059
Corticosteroid therapy	0.25 (0.06–1.10)	0.068	0.18 (0.04–0.79)	0.022	0.23 (0.05–1.02)	0.053

*Note*: The *p* values in bold are statistically significant (<0.0083).

Abbreviations: ALC, absolute lymphocyte count; CI, confidence interval; CSP, cyclosporine; HR, hazard ratio; MMF, mycophenolate mofetil; MTX, methotrexate; rDRI, refined disease risk index.

**FIGURE 4:**
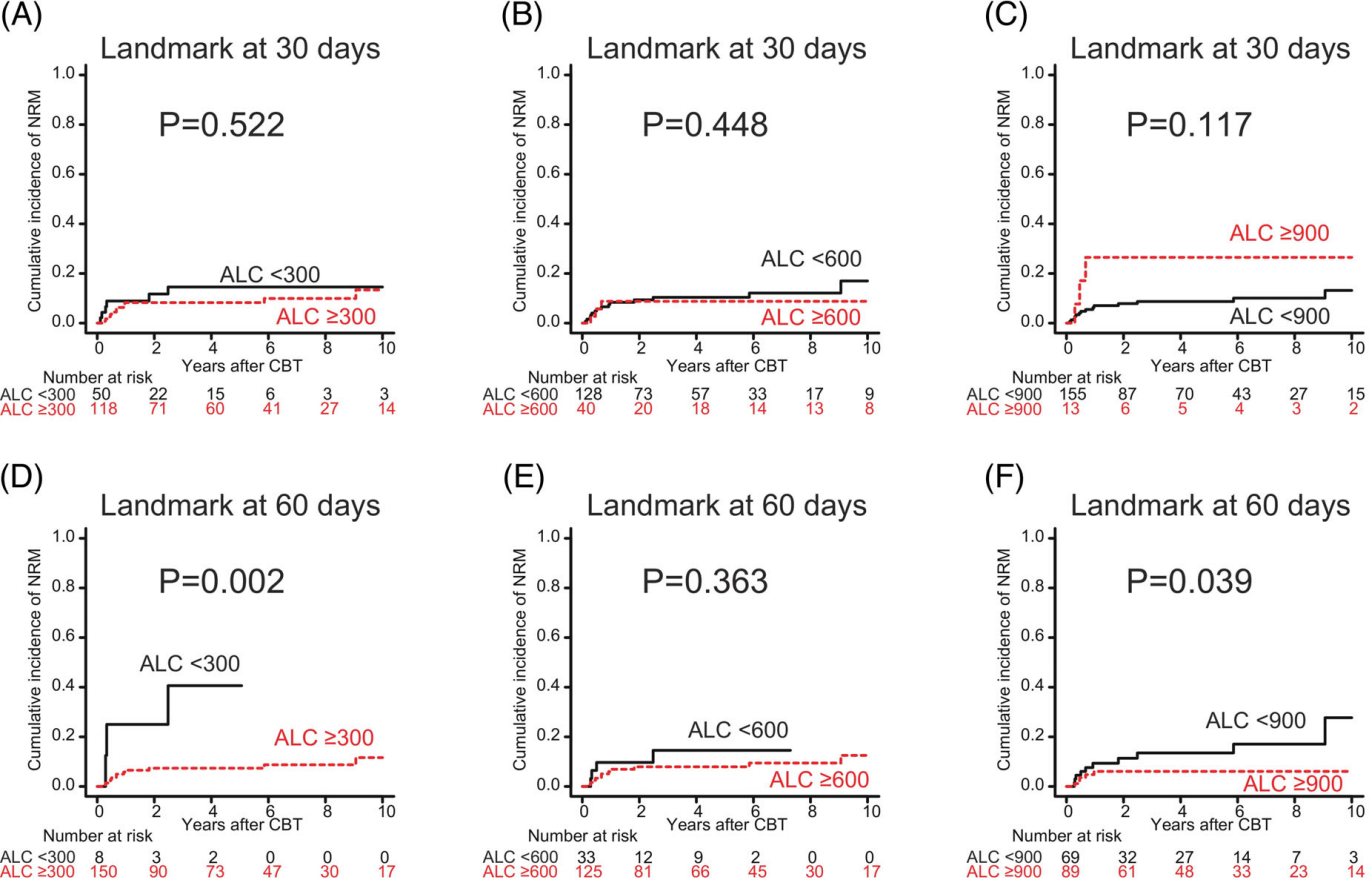
The cumulative incidence of non‐relapse mortality (NRM) following cord blood transplantation (CBT) according to absolute lymphocyte count (ALC) recovery of ≥300, ≥600, or ≥900/μl by 30 and 60 days. Cumulative incidence curves were plotted with a conditional landmark analysis at 30 days (A–C) and 60 days (D–F) following CBT

In the univariate analysis with a conditional landmark analysis at 60 days, ALC recovery ≥300/μl (*p* = 0.002) and ALC recovery ≥900/μl (*p* = 0.039) were significantly associated with lower NRM (Figure 4). In the multivariate analysis, only ALC recovery ≥300/μl by 60 days (HR: 0.15; 95% CI: 0.03–0.72; *p* = 0.018) was marginally associated with lower NRM (Table 5).

**TABLE 5:** Multivariable analysis for non‐relapse mortality

	ALC ≥300/μl		ALC ≥600/μl		ALC ≥900/μl	
	HR (95% CI)	*p* value	HR (95% CI)	*p* value	HR (95% CI)	*p* value
By 30 days						
ALC recovery	0.74 (0.22–2.45)	0.625	0.22 (0.04–1.21)	0.082	0.35 (0.05–2.08)	0.249
Age ≥45 years vs. <45 years	6.36 (1.23–32.84)	0.027	6.25 (1.21–32.35)	0.028	7.09 (1.36–36.84)	0.019
Female recipient vs. male recipient	0.09 (0.02–0.47)	**0.003**	0.06 (0.01–0.32)	**0.001**	0.06 (0.01–0.37)	**0.002**
Higher rDRI vs. lower rDRI	1.63 (0.62–4.24)	0.312	1.47 (0.55–3.88)	0.435	1.60 (0.61–4.17)	0.333
CD34^+^dose ≥1 × 10^5^/kg vs. <1 × 10^5^/kg	1.43 (0.49–4.16)	0.504	2.07 (0.70–6.14)	0.186	1.84 (0.60–5.58)	0.279
HLA disparities ≥3 vs. <3	0.82 (0.29–2.33)	0.717	0.67 (0.23–1.97)	0.472	0.76 (0.27–2.16)	0.614
CSP + MMF vs. CSP + MTX	3.42 (1.07–10.95)	0.037	3.40 (1.12–10.37)	0.030	3.80 (1.27–11.35)	0.016
Corticosteroid therapy	13.97 (5.04–38.73)	**<0.001**	23.42 (7.15–76.62)	**<0.001**	19.69 (6.02–64.34)	**<0.001**
By 60 days						
ALC recovery	0.15 (0.03–0.72)	0.018	1.04 (0.30–3.62)	0.942	0.37 (0.10–1.28)	0.117
Age ≥45 years vs. <45 years	6.33 (1.13–35.48)	0.035	5.43 (1.03–28.49)	0.045	3.70 (0.64–21.25)	0.142
Female recipient vs. male recipient	0.07 (0.01–0.44)	**0.004**	0.12 (0.02–0.63)	0.011	0.09 (0.01–0.49)	**0.004**
Higher rDRI vs. lower rDRI	1.67 (0.63–4.42)	0.302	1.46 (0.54–3.89)	0.446	1.23 (0.46–3.30)	0.670
CD34^+^dose ≥1 × 10^5^/kg vs. <1 × 10^5^/kg	2.00 (0.70–5.67)	0.192	1.63 (0.55–4.80)	0.369	2.22 (0.76–6.47)	0.143
HLA disparities ≥3 vs. <3	0.91 (0.31–2.68)	0.872	1.35 (0.49–3.76)	0.554	1.20 (0.43–3.30)	0.718
CSP + MMF vs. CSP + MTX	3.05 (0.94–9.92)	0.063	3.97 (1.25–12.57)	0.018	5.09 (1.49–17.38)	0.009
Corticosteroid therapy	7.96 (2.83–23.35)	**<0.001**	8.30 (2.99–23.00)	**<0.001**	8.54 (3.09–23.61)	**<0.001**

*Note*: The *p* values in bold are statistically significant (<0.0083).

Abbreviations: ALC, absolute lymphocyte count; CI, confidence interval; CSP, cyclosporine; HR, hazard ratio; MMF, mycophenolate mofetil; MTX, methotrexate; rDRI, refined disease risk index.

The causes of death in patients with or without ALC recovery ≥300/μl by 60 days are summarized in Table S3. Among causes of non‐relapse death, infection was not more common in patients without ALC recovery ≥300/μl by 60 days.

## 4 DISCUSSION

Immune reconstitution following allogeneic HCT is dependent upon the graft type. Indeed, lymphocyte recovery was slower in CBT recipients compared with lymphocyte recovery in matched unrelated adult donor recipients at early time‐points [15]. Most studies have evaluated the impact of ALC at 30–50 days even after CBT [1, 2, 3, 4]. However, our data showed that ALC values determined at 60 days could stratify survival and NRM following CBT. Therefore, the optimal time point to use ALC recovery as a prognostic tool following CBT could be later than the time points following allogeneic HCT from adult donors.

Corticosteroids, which are used to treat pre‐engraftment syndrome and GVHD, might affect ALC recovery following allogeneic HCT. However, most previous studies were unable to evaluate the confounding effects of corticosteroid therapy [1, 2, 3, 5, 6]. Although our multivariate analysis showed that corticosteroid therapy, which was treated as a time‐dependent covariate, was significantly associated with inferior OS and NRM using all thresholds and time points of ALC recovery, only ALC ≥300/μl at 60 days maintained their statistical significance for inferior OS following CBT. These data suggested that the association between ALC recovery and corticosteroid therapy might be important for assessing the prognostic impact of ALC recovery following HCT.

Our study had several limitations. First, it was a retrospective, single‐center study in Japan, and the number of patients involved was small. Therefore, our local clinical practice might have affected our results, which should therefore be interpreted with caution when applied to other cohorts receiving CBT. Second, the exact mechanisms underlying the association between improved ALC recovery and superior OS and NRM have not been fully elucidated. Higher CD34^+^ cell dose, which was significantly associated with better ALC recovery 300/μl at 60 days, could not affect the transplant outcomes after CBT in the multivariate analysis. Therefore, various posttransplant complications as well as cord blood unit selection could affect the ALC recovery. Further studies are needed to clarify these mechanisms. Third, we identified that the optimal prognostic threshold of ALC was 300/μl at 60 days after CBT, which is consistent with previous report in the bone marrow transplantation setting [6]. However, ALC ≥300/μl at 60 days was not associated with incidences of acute GVHD, infectious complications up to 60 days after CBT, probably because of the small number of patients without ALC ≥300/μl at 60 days. Therefore, the association between posttransplant complications and ALC recovery might be important for assessing the optimal prognostic threshold of ALC following CBT. Further studies are required to validate this threshold of ALC as a prognostic indicator after CBT.

In summary, our data clearly demonstrated the optimal prognostic threshold of ALC as 300/μl at 60 days following CBT, which was associated with OS and NRM following CBT. Although ALC recovery in routine peripheral blood analysis is a practical and easily evaluable method to measure immune reconstitution and to predict outcomes following CBT, further studies are warranted to evaluate the optimal time and threshold of ALC recovery as a prognostic tool following CBT.

## CONFLICT OF INTEREST

The authors declare no competing financial interests.

## AUTHOR CONTRIBUTIONS

Takaaki Konuma conceived the project, designed the research, analyzed the data, and wrote the paper. Yasuhito Nannya contributed to the critical review of the manuscript. All the other authors participated in the treatment of the patients and acquired the clinical data. All authors approved the final version.

## Supporting information

Supporting information

Supporting information
