# Supplementary figures and images for: Optimal time and threshold of absolute lymphocyte count recovery as a prognostic factor after single‐unit cord blood transplantation in adults
Source: EJHaem. 2021 Dec 29;3(1):191–8. doi: 10.1002/jha2.372 (PMC9176115; doi:10.1002/jha2.372)

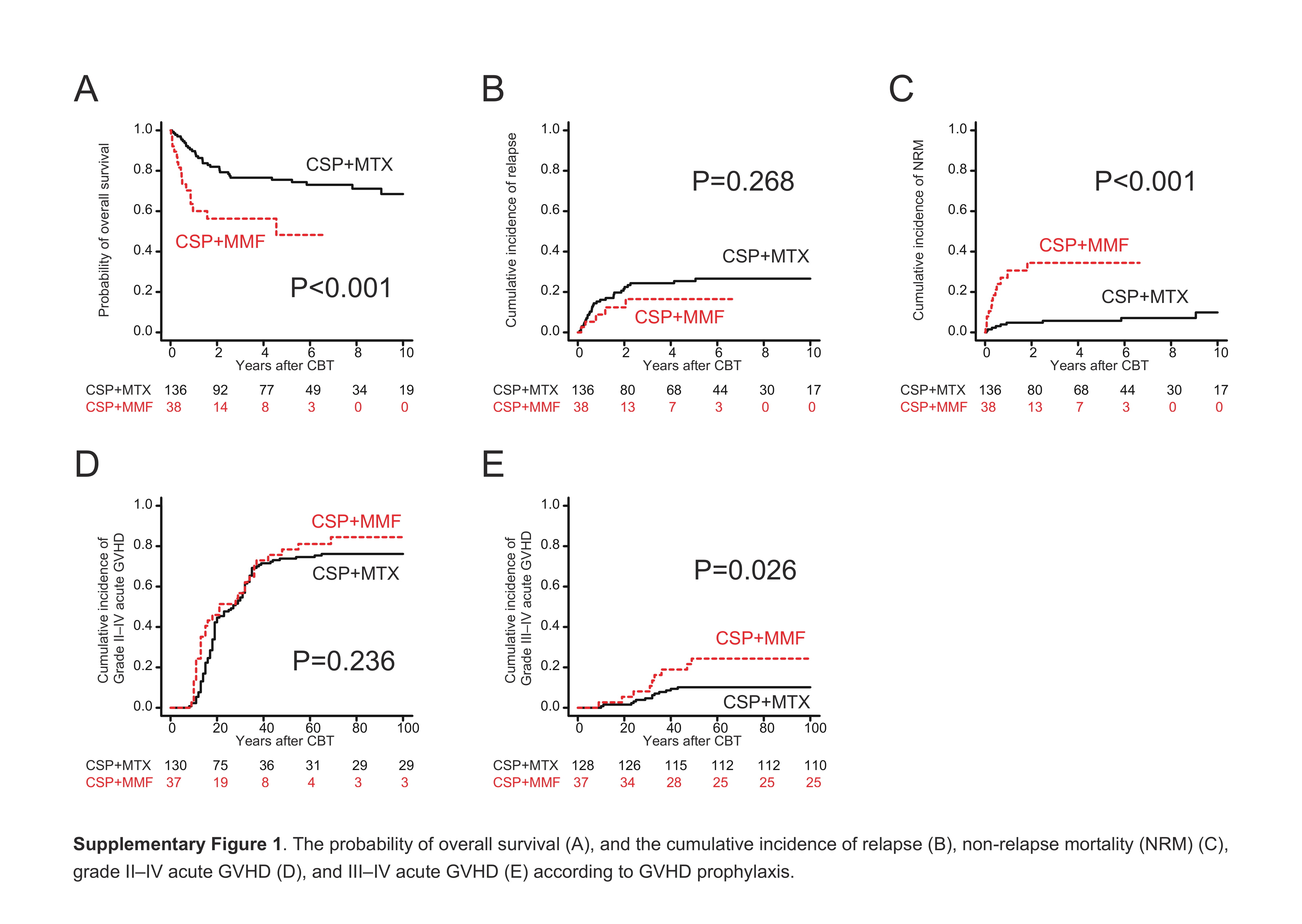

Supplement: Supplementary file 1 — Supporting information [file JHA2-3-191-s002.jpg]
