# Supplementary material for: Optimal time and threshold of absolute lymphocyte count recovery as a prognostic factor after single‐unit cord blood transplantation in adults
Source: EJHaem. 2021 Dec 29;3(1):191–8. doi: 10.1002/jha2.372 (PMC9176115; doi:10.1002/jha2.372)
Supplement: Supplementary file 2 — Supporting information [file JHA2-3-191-s001.docx]

**Supplementary Table 1**. Multivariable analysis for ALC recovery.

|  | ALC ≥300 /μL |  | ALC ≥600 /μL |  | ALC ≥900 /μL |  |
| --- | --- | --- | --- | --- | --- | --- |
|  | Adjusted HR (95%CI) | *P* value | Adjusted HR (95%CI) | *P* value | Adjusted HR (95%CI) | *P* value |
| By 30 days |  |  |  |  |  |  |
|  |  |  |  |  |  |  |
| TNC dose ≥2.0×10^7^/kg vs <2.0×10^7^/kg | 1.05 (0.66-1.65) | 0.820 | 1.09 (0.45-2.63) | 0.840 | 0.71 (0.17-2.91) | 0.630 |
| TNC dose ≥2.5×10^7^/kg vs <2.5×10^7^/kg | 1.35 (0.93-1.96) | 0.100 | 1.97 (0.95-4.07) | 0.066 | 3.67 (0.82-16.27) | 0.087 |
| TNC dose ≥3.0×10^7^/kg vs <3.0×10^7^/kg | 1.50 (1.04-2.16) | 0.029 | 1.83 (0.96-3.48) | 0.065 | 2.03 (0.69-6.00) | 0.200 |
|  |  |  |  |  |  |  |
| By 60 days |  |  |  |  |  |  |
|  |  |  |  |  |  |  |
| TNC dose ≥2.0×10^7^/kg vs <2.0×10^7^/kg | 0.98 (0.68-1.39) | 0.920 | 0.89 (0.54-1.47) | 0.660 | 1.17 (0.63-2.16) | 0.600 |
| TNC dose ≥2.5×10^7^/kg vs <2.5×10^7^/kg | 1.09 (0.80-1.49) | 0.570 | 1.09 (0.76-1.54) | 0.630 | 1.42 (0.93-2.18) | 0.100 |
| TNC dose ≥3.0×10^7^/kg vs <3.0×10^7^/kg | 1.31 (0.92-1.86) | 0.120 | 1.13 (0.78-1.65) | 0.510 | 1.17 (0.74-1.85) | 0.490 |

ALC, absolute lymphocyte count; TNC, total nucleated cell; HR, hazard ratio; CI, confidence interval.

HRs and P values for each TNC dose were adjusted for age (<45 vs. ≥45 years), recipient sex (male vs. female), disease risk index (low/intermediate vs. high/very high), HLA disparities defined as a high-resolution for HLA-A, -B, and -DRB1 (<3 vs. ≥3), and GVHD prophylaxis (cyclosporine and methotrexate vs. cyclosporine and mycophenolate mofetil).

**Supplementary Table 2**. Incidences of GVHD and infectious complication by 60 days according to ALC recovery.

|  |  | Grade II–IV aGVHD |  | Grade III–IV aGVHD |  | CMV antigenemia |  | Virus infections |  | Bacteremia |  |
| --- | --- | --- | --- | --- | --- | --- | --- | --- | --- | --- | --- |
| By 60 days | Numbers |  | *P* value |  | *P* value |  | *P* value |  | *P* value |  | *P* value |
| ALC ≥300 /μL | 155 | 119 (78%) | 1.000 | 19 (13%) | 0.080 | 106 (69%) | 0.437 | 11 (7%) | 0.465 | 21 (14%) | 0.314 |
| ALC <300 /μL | 8 | 6 (75%) |  | 3 (38%) |  | 7 (88%) |  | 1 (13%) |  | 2 (25%) |  |
|  |  |  |  |  |  |  |  |  |  |  |  |
| ALC ≥600 /μL | 128 | 99 (78%) | 1.000 | 13 (10%) | **0.018** | 84 (66%) | **0.035** | 8 (6%) | 0.698 | 16 (13%) | 0.401 |
| ALC <600 /μL | 33 | 26 (79%) |  | 9 (28%) |  | 28 (85%) |  | 3 (9%) |  | 6 (18%) |  |
|  |  |  |  |  |  |  |  |  |  |  |  |
| ALC ≥900 /μL | 92 | 75 (82%) | 0.176 | 9 (10%) | 0.109 | 58 (63%) | **0.040** | 4 (4%) | 0.208 | 10 (11%) | 0.254 |
| ALC <900 /μL | 69 | 50 (73%) |  | 13 (19%) |  | 54 (78%) |  | 7 (10%) |  | 12 (17%) |  |

GVHD, graft-versus-host disease; ALC, absolute lymphocyte count; CMV, cytomegalovirus.

The *P* values in bold are statistically significant (<0.05).

**Supplementary Table 3**. Cause of death according to absolute lymphocyte count (ALC) ≥300 /μL by 60 days after CBT.

|  | Total | ALC <300 /μL | ALC ≥300 /μL |
| --- | --- | --- | --- |
| Number of deaths | 43 | 5 | 38 |
| Relapse | 26 (60%) | 2 (40%) | 24 (63%) |
| Infection | 5 (12%) | 0 | 5 (13%) |
| GVHD | 6 (14%) | 1 (20%) | 5 (13%) |
| Organ failure | 3 (7%) | 2 (40%) | 1 (3%) |
| Second cancer | 1 (2%) | 0 | 1 (3%) |
| Others | 2 (5%) | 0 | 1. (5%) |

CBT, cord blood transplantation; GVHD, graft-versus-host disease.
